# Chronic inflammation mediates the relationship between physical activity and telomere length

**DOI:** 10.1007/s11357-025-01818-z

**Published:** 2025-08-05

**Authors:** Anamika Nanda, Daniel H. Aslan, M. Katherine Sayre, Pradyumna K. Bharadwaj, Madeline Ally, Hyun Song, Amit Arora, Silvio Maltagliati, Mark H. C. Lai, Rand R. Wilcox, Yann C. Klimentidis, Gene E. Alexander, David A. Raichlen

**Affiliations:** 1 Human and Evolutionary Biology Section, Department of Biological Sciences, University of Southern California, Los Angeles, CA USA; 2 Department of Anthropology, University of California Santa Barbara, Santa Barbara, CA USA; 3 Department of Psychology, University of Arizona, Tucson, AZ USA; 4 Department of Epidemiology and Biostatistics, Mel and Enid Zuckerman College of Public Health, University of Arizona, Tucson, AZ USA; 5 Department of Psychology, University of Southern California, Los Angeles, CA USA; 6 BIO5 Institute, University of Arizona, Tucson, AZ USA; 7 Evelyn F. McKnight Brain Institute, University of Arizona, Tucson, AZ USA; 8 Department of Psychiatry, University of Arizona, Tucson, AZ USA; 9 Neuroscience Graduate Interdisciplinary Program, University of Arizona, Tucson, AZ USA; 10 Physiological Sciences Graduate Interdisciplinary Program, University of Arizona, Tucson, AZ USA; 11 Arizona Alzheimer’s Consortium, Phoenix, AZ USA; 12 Department of Anthropology, University of Southern California, Los Angeles, CA USA

**Keywords:** C-reactive protein, Epidemiology, Physical activity, Telomere length, UK Biobank

## Abstract

A physically active lifestyle benefits cellular aging, however the mechanisms linking physical activity (PA) with longevity remain unclear. PA is associated with longer telomere length (TL), while shorter TL has been associated with increased cellular aging. Some research suggests increased levels of inflammatory markers, such as C-reactive protein (CRP), are associated with telomere dysfunction. We tested the hypothesis that CRP levels mediate the association between PA and TL. Using data from the UK Biobank, we analyzed adjusted leukocyte T/S ratio (relative telomere to single gene copy), serum CRP, and moderate-to-vigorous physical activity (MVPA) data via device-measured actigraphy. We applied general linear regressions and a causal mediation analysis with 10,000 bootstraps while controlling for a range of covariates (age, BMI, smoking status, sex, ethnicity, time between data collection, time wearing the accelerometer, and the Townsend Deprivation Index). Variables of interest were transformed to approximate normality. A total of 79,873 participants were included in the final analytic sample. MVPA and CRP were both significant predictors of TL (β_MVPA_ = 3.03e − 03 [95%CI = 1.58e − 03, 4.47e − 03], p_MVPA_ = 4.10e − 05; β_CRP_ =  − 1.36e − 03 [95%CI =  − 1.87e − 03, − 8.40e − 04], p_CRP_ = 2.52e − 07, respectively). The association between MVPA and TL was mediated by CRP, accounting for 8.65% [95% CI: 4.77%, 16.0%] of the total effect (β [95%CI] = 3.31e − 03 [1.84e − 03, 4.75e − 03], *p* < 2e − 16). Our analysis supports the hypothesis that CRP mediates the relationship between MVPA and TL. These novel findings suggest a potential pathway where PA is associated with lower CRP concentrations, which in turn is associated with longer average TL.

**Graphical abstract:**

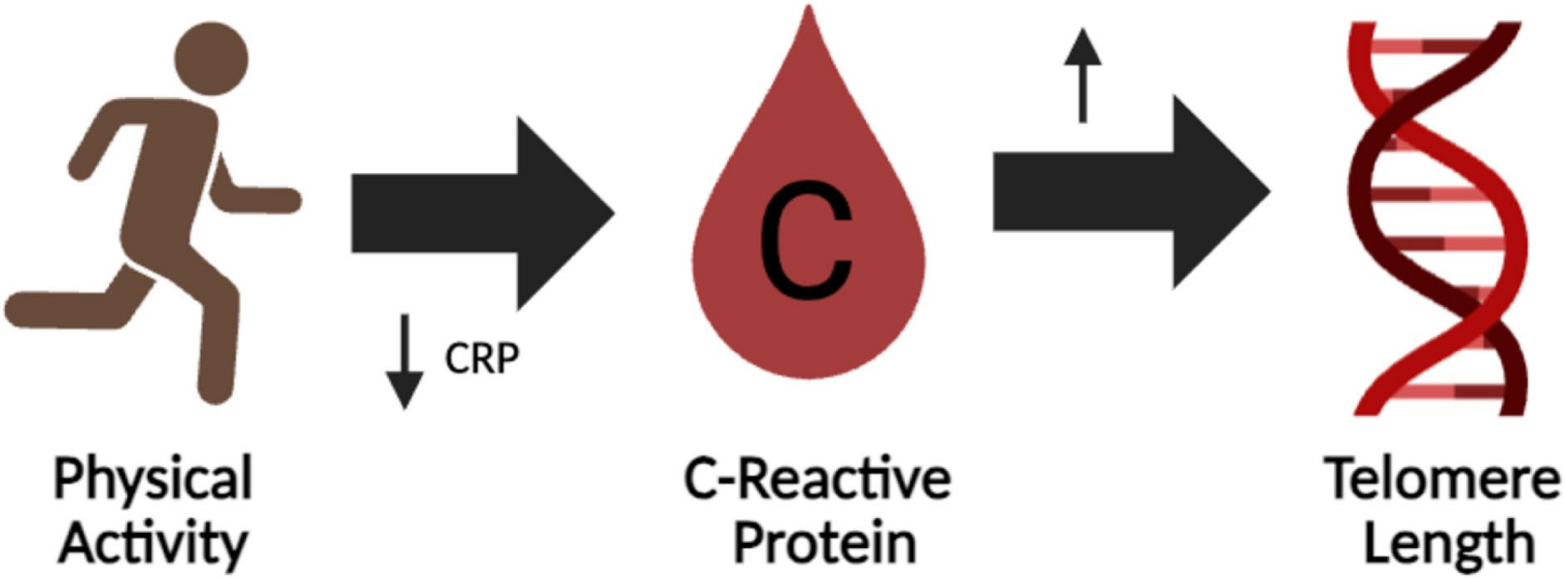

**Supplementary Information:**

The online version contains supplementary material available at 10.1007/s11357-025-01818-z.

## Introduction

Physical activity (PA) is beneficial to our health and is associated with longer lifespan in several cohort studies [1, 2]. One potential mechanism relating PA and lifespan is through impacts on cellular aging. Recent work suggests PA decreases the attrition rate of the ends of our chromosomal caps, known as telomeres [3, 4]. Telomeres are repeating strings of DNA nucleotides that shorten with age and cellular division [5]. Telomere length (TL) is used as a biomarker of cellular aging, and decreased TL is associated with cellular senescence [6–8]. Although the relationship between PA and TL varies by PA intensity, PA duration, and participants’ demographics (e.g., age), generally individuals who engage in moderate intensity PA have longer TL compared to less active individuals [9–12]. Thus, PA affects telomere attrition, providing a connection between PA and lifespan. However, the underlying mechanisms explaining the relationship between PA and cellular aging remain unclear.

One possible pathway linking PA and TL is through inflammatory processes [10]. Shorter TL is associated with higher risk of inflammatory diseases, including ulcerative colitis, aplastic anemia, as well as a variety of cancers, which often develop in inflammatory environments [13]. Inflammation and telomere deterioration are involved in a feedback loop where inflammation drives telomere dysfunction, and telomeres regulate inflammatory responses [14]. Further, inflammation can cause excessive oxidative stress which is linked to telomere deterioration as well [15]. PA is a well-established modulator of chronic inflammation [16]. Regular high-intensity PA can manage the strength and duration of inflammatory responses, potentially leading to a healthier phenotype characterized by slower cellular aging, longer TL, and reduced risks for chronic diseases linked with mortality [17, 18]. One of the key inflammatory markers, C-reactive protein (CRP), plays a crucial role in this process. CRP is secreted by the liver in response to inflammation in the body [19, 20]. While CRP levels can indicate acute levels of systemic inflammation, it is often considered a reliable measure of chronic inflammation [21]. Like other inflammatory cytokines, increased levels of CRP are associated with shorter TL [22], and lower levels of CRP are associated with longer TL [23]. Further, moderate levels of PA are associated with reduced CRP levels [24] through both direct and indirect effects (e.g., reducing cytokine production in fat and muscle and reducing body fat) [15].

While previous work suggests PA and TL may be linked through the effects of PA on inflammation, no work to date has directly examined the potential for chronic inflammation to mediate the relationship between PA and TL. Here, using data from the UK Biobank, we first examine relationships among CRP, PA, and TL in a sample of middle-age and older adults. Next, we use a causal mediation analysis to determine whether CRP mediates the relationship between PA and TL. We hypothesize that greater time spent in PA will be associated with longer TL, and that lower CRP concentrations will mediate this relationship.

## Methods

### Study design and participants

We analyzed data from the UK Biobank (https://www.ukbiobank.ac.uk/), a longitudinal large-scale cohort that includes over 500,000 men and women aged 39 to 71 years with baseline visits from 2006 to 2010 [25]. All participants provided written informed consent, and study approval was obtained from the National Health Service and the National Research Ethics Service [26]. Upon joining the study, participants answered touchscreen questionnaires including information on anthropometric, lifestyle, medical, demographic information, and donated blood samples. Objective PA measures were determined within a sub-study from 2013 to 2015 with a total of 103,684 participants who agreed to wear a three-axis logging accelerometer (AX3; Axivity) for 24 hours per day for 7 days on their dominant wrist [27].

### Accelerometer data

Moderate-to-vigorous physical activity (MVPA) was identified from raw accelerometer data using a previously published machine learning algorithm developed and validated for use with the UK Biobank [27]. The algorithm was developed and validated by Walmsley et al. [28] in a separate study in which 152 adults from 18 to 91 years old wore an AX3 accelerometer, a wearable camera while awake, and maintained an activity diary. Annotators used the camera footage and activity diary information to classify the accelerometer data with labels from the Compendium of Physical Activities [29] and trained machine-learning models to classify behaviors in 30-second time windows of accelerometer data [27]. To be included in our study, accelerometer data needed to pass the UK Biobank calibration QC. Further, individuals needed to have 3 or more days of data, data in each hour of 24-hour days, and average acceleration magnitudes less than 100 mg. All accelerometer data was collected from 2013 to 2015 [30].

### Blood measures: C-reactive protein and telomere length

CRP (mg/L) levels were determined using immunoturbidimetric-high sensitivity analysis on a Beckman Coulter AU5800 in mg/L [31]. Methods for blood collection and analyses have been described previously [32]. Baseline blood samples were collected from 2006 to 2010 [25] and processed with an extensively tested protocol [32]. CRP assay quality control procedures from the UK biobank can be found in detail at: https://biobank.ctsu.ox.ac.uk/ukb/refer.cgi?id=5636 [33].

Adjusted leukocyte T/S ratio (relative telomere to single gene copy) were used in our analyses. TL was derived from DNA samples of peripheral blood leukocyte samples, and multiplex quantitative polymerase chain reactions (qPCR) was used to measure the ratio of telomere repeat copy number (T) relative to that of a simple copy gene (S) [34]. Approximately 14,326 samples were invalid, failing leukocyte TL assay quality control as described in Codd et al. [34].

### Covariates

Participants reported their age at the baseline visit, and sex was assessed by participants identifying themselves as ‘male’ or ‘female’. Time between baseline blood collection and PA data collection was measured in years. Other covariates included in statistical analyses were ethnicity (white vs. non-white), time spent wearing the accelerometer in days, body mass index, received a chronic disease diagnosis of cardiovascular disease, diabetes, or cancer (0 for ‘no chronic diseases’, 1 for ‘present’), and self-reported smoking status (0 for ‘prefer not to answer’, 1 for ‘never’, 2 for ‘previous’, and 3 for ‘currently’). Finally, we included Townsend Deprivation Index, a census-based proxy measure for socioeconomic status composed of data on car ownership, household overcrowding, household owner-occupation, and unemployment [35].

### Statistical analysis

T/S and MVPA measures were transformed to approximate normal distributions by taking their square root, and CRP was log transformed to approximate a normal distribution [36]. Three fully adjusted general linear regression models were conducted in total. The first model included TL as the dependent variable and MVPA as the independent variable, controlling for covariates. The second model included CRP as the dependent variable and MVPA as the independent variable, controlling for covariates. Finally, the third model examined the relationship between TL and MVPA controlling for CRP as well as the other covariates. Mediation analysis assumptions were met, and collinearity was assessed prior to data analysis [37]. The mediation models were performed using bootstrap resampling with 10,000 iterations to produce 95% confidence intervals with the percentile method [38] using the ‘mediation’ package (V.4.5.0) in R (V.4.31) [39], with MVPA as the independent variable, TL as the dependent variable, and CRP as the mediating variable. Minimally adjusted models were first performed to control for sex and age at baseline visit. Subsequently, fully adjusted models were run including all covariates listed above. Finally, sensitivity analyses were performed to assess robustness of the main results focusing on adults older than 60 years of age, as well as analyses stratified by sex. Identical general linear regression and a bootstrapped causal mediation analysis (10,000 simulations) were run controlling for the covariates listed above.

## Results

**Fig. 1:**
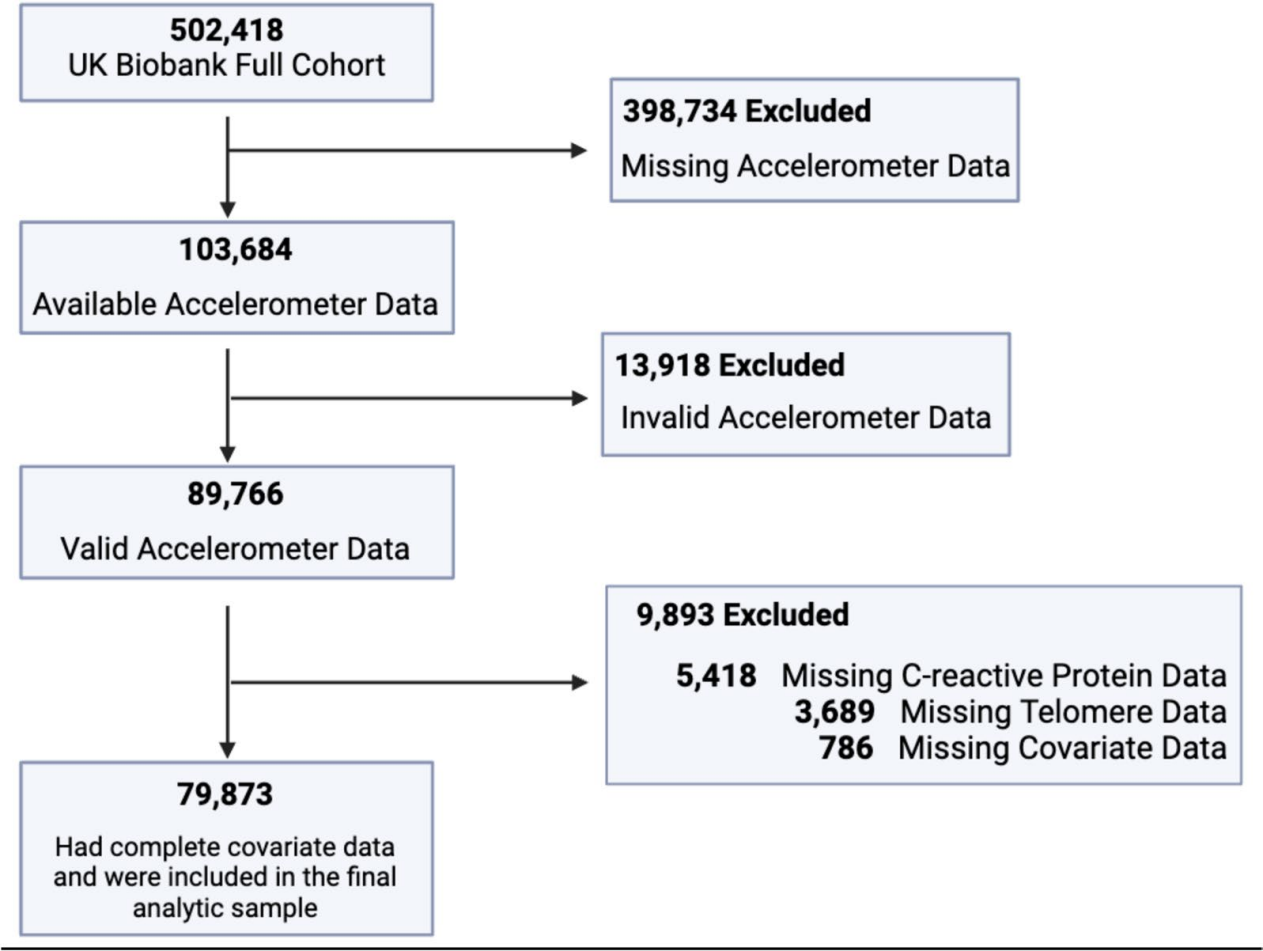
Flow of study participants

A total of 79,873 participants who had valid and complete covariate data were included in the final analytic sample (see Fig. 1). Average age was 56.69 years (SD 7.81), participants were predominantly white (96.99%), and 44,836 participants reported as female (56.13%). Participant demographics appear in Table 1.

**Table 1:** Participant demographics

Characteristic	Female (*n* = 44,836)	Male (*n* = 35,037)	Total (*n* = 79,873)
		Mean (SD) or *n* (%)	
Ethnicity (% white)	43,428 (96.86)	34,042 (97.16)	77,470 (96.99)
Smoking status (% never smoked)	27,376 (61.06)	18,259 (52.11)	45,635 (57.13)
Chronic diseases (% without)	32,293 (72.02)	22,646 (64.63)	54,939 (68.78)
Baseline age (yrs)	56.18 (7.72)	57.35 (7.88)	56.69 (7.81)
BMI	26.27 (4.84)	27.27 (4.00)	26.71 (4.52)
Date difference from samples (yrs)	5.69 (1.07)	5.70 (1.07)	5.69 (1.07)
Actigraph wear time (days)	6.70 (0.54)	6.73 (6.55)	6.72 (0.55)
Townsend deprevation index	−1.70 (2.81)	−1.79 (2.81)	−1.74 (2.81)
Adjusted T.S ratio	0.85 (0.13)	0.82 (0.13)	0.84 (0.13)
CRP (mg/L)	2.33 (3.93)	2.12 (3.85)	2.24 (3.89)
MVPA (hrs/day)	0.59 (0.51)	0.83 (0.66)	0.70 (0.60)

In the minimally adjusted regression models, time spent engaged in MVPA was positively associated with TL (β [95%CI] = 4.30e − 03 [2.94e − 03, 5.72e − 03], *p* = 9.34e − 10). Higher levels of MVPA were significantly associated with lower CRP concentrations (β [95%CI] =  − 0.563 [− 0.584, − 0.543], *p* < 2e − 16). In a regression including MVPA and CRP, both were significant predictors of TL (β_MVPA_ [95%CI] = 3.36e − 03 [1.95e − 03, 4.77e − 03], p_MVPA_ = 3.12e − 06; β_CRP_ [95%CI] =  − 1.73e − 03 [− 2.20e − 03, − 1.27e − 03], p_CRP_ = 3.51e − 13 respectively). In the minimally adjusted causal mediation analysis, CRP partially mediated the relationship between MVPA and TL, accounting for 22.46% [95%CI: 14.66%, 35.59%] of the total effect (β [95%CI] = 4.33e − 03 [2.92e − 03, 5.71e − 03], *p *< 2e − 16). There was a significant indirect effect of MVPA on TL through CRP (β [95%CI] = 9.73e − 04 [7.07e − 04, 1.24e − 03], *p* < 2e − 16), and direct effect of MVPA on TL (β [95%CI] = 3.35e − 03 [1.92e − 03, 4.78e − 03], *p* < 2e − 16).

**Fig. 2:**
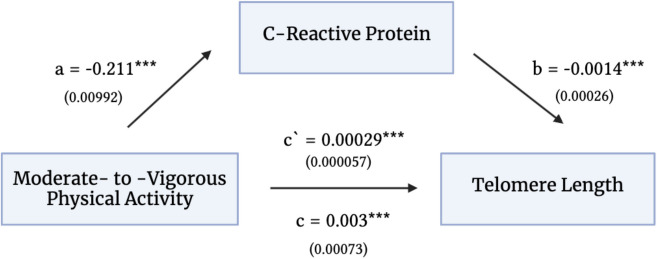
Mediation model. Values are β-coefficients (standard error). In this figure, c is the direct effect of moderate-to-vigorous physical activity on telomere length and c’ is the indirect effect of moderate-to-vigorous physical activity on telomere length through c-reactive protein. Statistical significance is noted by ***, which indicates *p* < 0.001. Models were adjusted for the following covariates: sex, age at baseline visit, time between baseline blood collection and moderate-to-vigorous physical activity data, ethnicity, wear time of accelerometer, body mass index, presence of chronic disease, self-reported smoking status, and socioeconomic status

When including all covariates in the fully adjusted model (see methods), there was a positive relationship between time spent engaged in MVPA and TL (β [95%CI] = 3.31e − 03 [1.87e − 03, 0.005], *p* = 6.77e − 06). MVPA was significantly associated with CRP (β [95%CI] =  − 0.211 [− 0.23, − 0.19], *p* < 2e − 16). When both were included in the model, MVPA and CRP were significant predictors of TL (β_MVPA_ [95%CI] = 3.03e − 03 [1.58e − 03, 4.47e − 03], p_MVPA_ = 4.10e − 05; β_CRP_ [95%CI] =  − 1.36e − 03 [− 1.87e − 03, − 8.40e − 04], p_CRP_ = 2.52e − 07 respectively). The association between MVPA and TL was significantly partially mediated by CRP (Fig. 2), with the overall proportion mediated accounting for 8.65% [95% CI: 4.77%, 16.0%] of the total effect (β [95%CI] = 3.31e − 03 [1.84e − 03, 4.75e − 03], *p* < 2e − 16). There was a significant indirect effect of MVPA on TL through CRP (β [95%CI] = 2.85e − 04 [1.73e − 04, 4.00e − 04], *p* < 2e − 16), and direct effect of MVPA on TL (β [95% CI] = 3.02e − 03 [1.56e − 03, 4.47e − 03], *p* = 2e − 04). In our sensitivity analysis including only participants > 60 years of age (*n* = 47,658), female participants (*n* = 44,836), and male participants (*n* = 35,037) results did not differ substantially from the main analyses (see eTables 1–6 in supplement 1).

## Discussion

Within the UK Biobank cohort, we found that both MVPA and CRP were significantly associated with TL. In both the minimally adjusted and fully adjusted models, MVPA was positively associated with TL, and in turn, CRP was negatively associated with TL. Our results suggest that inflammation plays a significant role in partially mediating the relationship between MVPA and TL. The results from our sensitivity analysis focusing on adults older than 60 years old supported findings from our main analysis. These findings build on previous work linking PA and lifespan across several cohorts [1–3, 10–13] and suggest that one potential mechanism relating PA and cellular senescence is through the association between PA and chronic inflammation. Since our analysis found a partial mediation effect, there are likely other physiological mechanisms linking PA and TL, possibly through decreasing cortisol and psychosocial stress [40, 41], however, chronic inflammation may be an important aspect of this relationship.

Extensive research supports the hypothesis that PA reduces overall levels of inflammation [23, 37, 38]. However, inflammatory cytokines and proteins can have different roles and responses to PA [42, 43]. Specifically, the strength of the inflammatory reaction to PA can vary across cytokines. PA plays an important role in regulating systemic inflammation in both acute and chronic circumstances [44]. Single bouts of PA can induce a temporary pro-inflammatory environment, characterized by an increase in circulating levels of skeletal muscle-derived IL-6 [43, 45]. This short-term acute increase in inflammation may be linked with muscle repair [44, 45]. In contrast, long-term participation in PA is associated with lower levels of pro-inflammatory biomarkers (i.e. reduced levels TNF-$\alpha$ and CRP) [46–48]. Although PA can increase both pro- and anti-inflammatory cytokines, long-term PA is likely associated with lower chronic inflammation, possibly due to reductions in visceral fat and the stimulation of cortisol and adrenaline production [46, 49, 50]. While the duration, intensity, and frequency of PA influence the nature of the inflammatory response—whether acute or chronic—extensive evidence supports the overall anti-inflammatory effects of long-term PA.

Although short-term PA interventions have not led to significant TL increases compared to inactive control groups [9], chronic PA-induced reductions in inflammation may play a crucial role in preserving TL. Recent work suggests that reducing chronic inflammation offers protective benefits for TL [14, 51], and prior work has found evidence of inflammation playing a role in TL in nonhuman animal models. For example, studies in mouse models show that chronic inflammation induces telomere dysfunction [52]. One mechanism linking chronic inflammation to TL attrition is through inhibition of telomerase, an enzyme that maintains TL [53]. Pro-inflammatory cytokines like IL-6 and TNF-$\alpha$ can inhibit telomerase by activating the NF-kB pathway, accelerating telomere shortening [54]. PA, which reduces chronic inflammation, may help preserve TL [14]. Our results are in line with these mechanisms and suggest that CRP plays a significant role in mediating the relationship between MVPA and TL. Other mechanisms that may also mediate the relationship between PA and TL include psychosocial stress and cortisol [41, 55]. PA has been associated with both lower cortisol levels [56] and elevated mood-related neurotransmitter activity [57] via modulation of the hypothalamic–pituitary–adrenal axis [56, 58]. In turn, reductions in psychosocial stress are associated with longer TL [59], a relationship that may be related with cortisol given how the hormone is associated with shorter TL [60].

While this study has several strengths, including a large sample size and a wide array of covariates, there are also limitations. First, we included CRP as the only inflammatory biomarker. It is possible that other inflammatory cytokines play a role in the relationship between PA and TL, however we were limited by the availability of biomarkers in the UK Biobank dataset. The inflammatory response involves several cytokines and measuring more inflammatory biomarkers would provide a holistic perspective on the role of inflammation in links between PA and TL attrition. Second, our study includes data collected at different time points, and our methods rely on the assumption that PA was stable from baseline to accelerometer measurement. Although prior work has validated the longitudinal reproducibility of the accelerometer-derived PA measures in the UK Biobank [61], and we controlled for the time difference between blood and accelerometer sampling, it is still possible that this time difference may impact interpretations of our results. Third, much of this dataset represented a white and older demographic, and future studies should determine whether these results are reflected in a more ethnically diverse and younger population. Finally, as an observational study, we are unable to determine causal links between PA, inflammation, and TL. Future work focused on long-term randomized controlled trials is needed to determine causality.

In conclusion, our results suggest that inflammation partially mediates the relationship between MVPA and TL. Time spent engaging in MVPA was associated with lower CRP concentrations, which in turn was associated with longer average TL. While chronic inflammation, as measured by CRP, partially mediates the relationship between MVPA and TL, other inflammatory markers, or other physiological mechanisms are likely involved in the relationship between PA and cellular aging. Further research is needed to determine whether other inflammatory biomarkers are involved in this mechanism.

## Supplementary Information

Below is the link to the electronic supplementary material.

Supplementary file1 (DOCX 384 KB)

## Data Availability

Data used in this project are available via application UK Biobank.
