# Supplementary material for: Chronic inflammation mediates the relationship between physical activity and telomere length
Source: GeroScience. 2025 Aug 5;48(2):2863–71. doi: 10.1007/s11357-025-01818-z (PMC12972198; doi:10.1007/s11357-025-01818-z)
Supplement: Supplementary file 1 — Supplementary file1 (DOCX 384 KB) [file 11357_2025_1818_MOESM1_ESM.docx]

**Supplementary Tables**

Intended for publication as an online data supplement
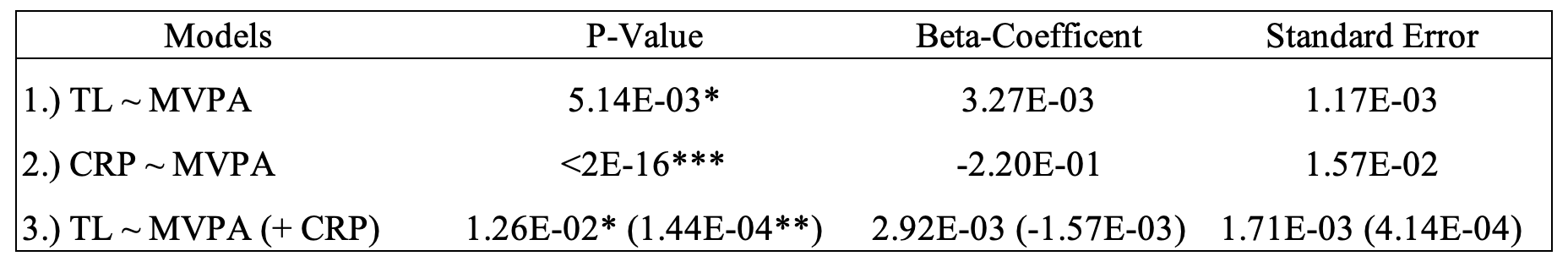


eTable 1. Regression models including moderate-to-vigorous physical activity (MVPA), C-reactive protein (CRP), and telomere length (TL) results for sensitivity analysis focused on participants > 60 years of age. Statistical significance is noted by * p <0.05, ** p <0.005, ***p <0.0001.


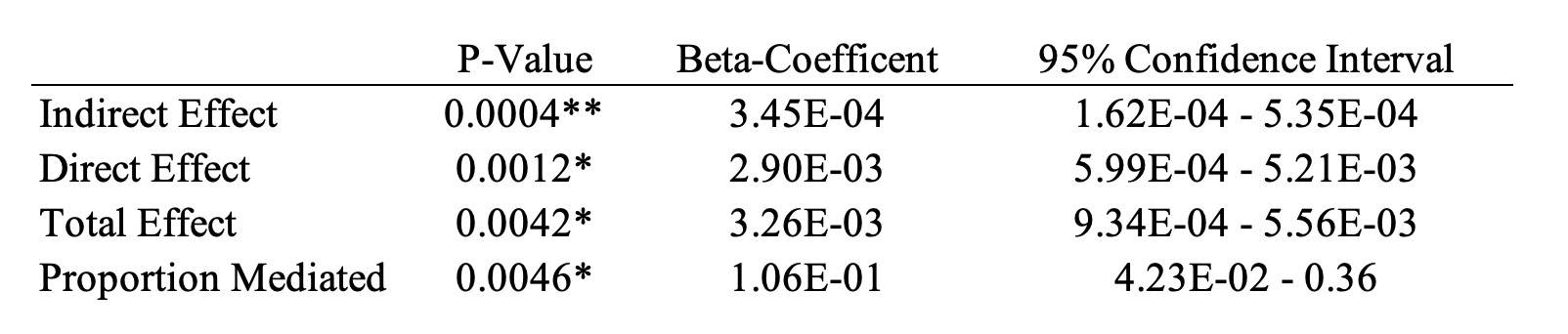


eTable 2. Causal mediation results between moderate-to-vigorous physical activity (MVPA), C-reactive protein (CRP), and telomere length (TL) sensitivity analysis focused on participants > 60 years of age. Statistical significance is noted by * p <0.05, ** p <0.001, ***p <0.0001.


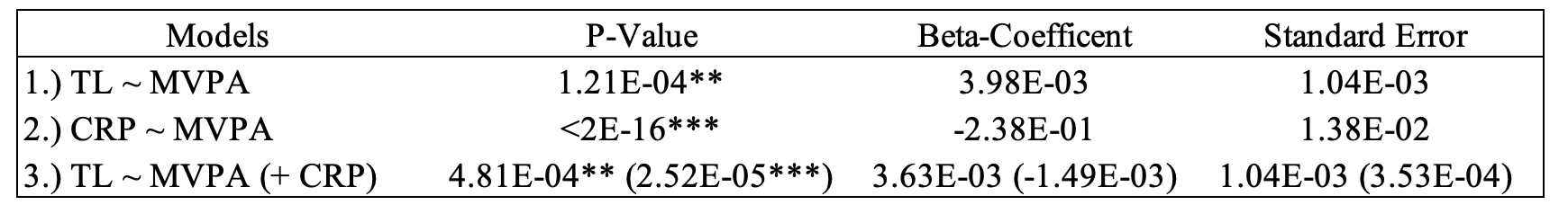


eTable 3. Regression models including moderate-to-vigorous physical activity (MVPA), C-reactive protein (CRP), and telomere length (TL) results for sensitivity analysis focused on female participants. Statistical significance is noted by * p <0.05, ** p <0.001, ***p <0.0001.


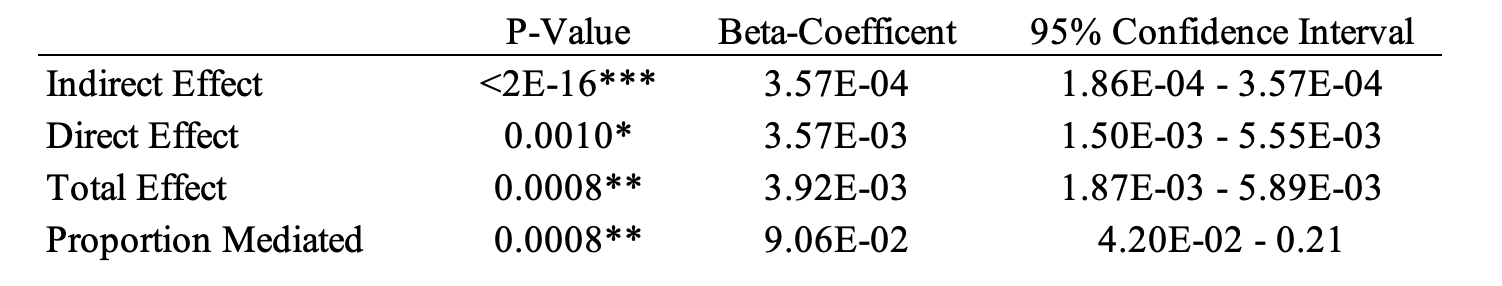


eTable 4. Causal mediation results between moderate-to-vigorous physical activity (MVPA), C-reactive protein (CRP), and telomere length (TL) sensitivity analysis focused on female participants. Statistical significance is noted by * p <0.05, ** p <0.001, ***p <0.0001.


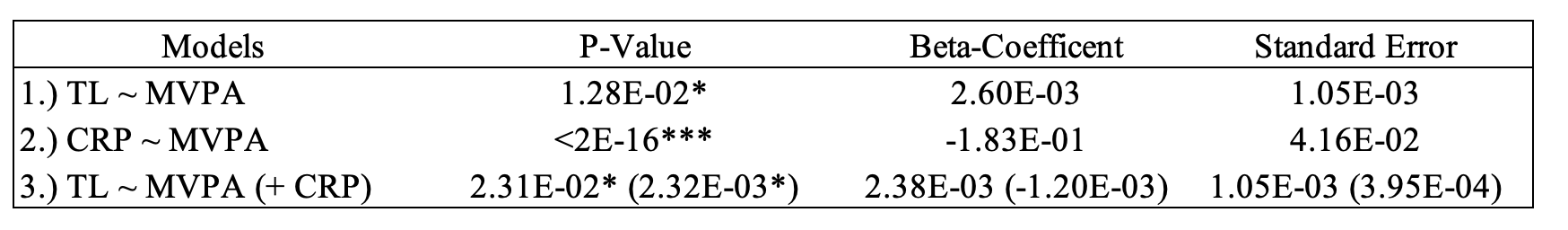


eTable 5. Regression models including moderate-to-vigorous physical activity (MVPA), C-reactive protein (CRP), and telomere length (TL) results for sensitivity analysis focused on male participants. Statistical significance is noted by * p <0.05, ** p <0.001, ***p <0.0001.


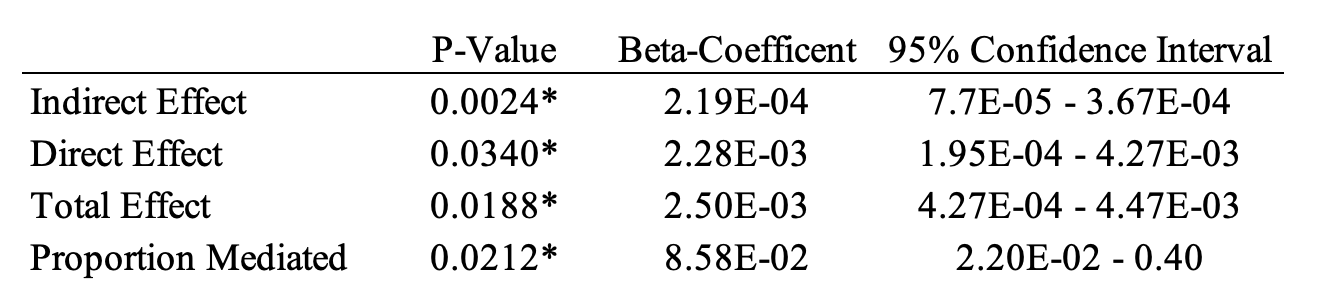


eTable 6. Causal mediation results between moderate-to-vigorous physical activity (MVPA), C-reactive protein (CRP), and telomere length (TL) sensitivity analysis focused on male participants. Statistical significance is noted by * p <0.05, ** p <0.001, ***p <0.0001.
